# Current Trends on the Involvement of Zinc, Copper, and Selenium in the Process of Hepatocarcinogenesis

**DOI:** 10.3390/nu16040472

**Published:** 2024-02-06

**Authors:** Takashi Himoto, Tsutomu Masaki

**Affiliations:** 1 Department of Medical Technology, Kagawa Prefectural University of Health Sciences, 281-1, Hara, Mure-cho, Takamatsu 761-0123, Kagawa, Japan; 2 Department of Gastroenterology and Neurology, Faculty of Medicine, Kagawa University, 1750-1, Ikenobe, Miki-cho 761-0793, Kagawa, Japan; tmasaki@med.kagawa-u.ac.jp

**Keywords:** hepatocellular carcinoma, zinc, copper, selenium, antitumor effect, chemosensitivity, nanoparticles

## Abstract

Numerous nutritional factors increase the risk of hepatocellular carcinoma (HCC) development. The dysregulation of zinc, copper, and selenium homeostasis is associated with the occurrence of HCC. The impairment of the homeostasis of these essential trace elements results in oxidative stress, DNA damage, cell cycle progression, and angiogenesis, finally leading to hepatocarcinogenesis. These essential trace elements can affect the microenvironment in HCC. The carrier proteins for zinc and copper and selenium-containing enzymes play important roles in the prevention or progression of HCC. These trace elements enhance or alleviate the chemosensitivity of anticancer agents in patients with HCC. The zinc, copper, or selenium may affect the homeostasis of other trace elements with each other. Novel types of cell death including ferropotosis and cupropotosis are also associated with hepatocarcinogenesis. Therapeutic strategies for HCC that target these carrier proteins for zinc and copper or selenium-containing enzymes have been developed in in vitro and in vivo studies. The use of zinc-, copper- or selenium-nanoparticles has been considered as novel therapeutic agents for HCC. These results indicate that zinc, copper, and selenium may become promising therapeutic targets in patients with HCC. The clinical application of these agents is an urgent unmet requirement. This review article highlights the correlation between the dysregulation of the homeostasis of these essential trace elements and the development of HCC and summarizes the current trends on the roles of these essential trace elements in the pathogenesis of hepatocarcinogenesis.

## 1. Introduction

It is well-established that hepatocellular carcinoma (HCC) is one of the most common malignancies worldwide. According to current epidemiological studies, HCC is the fourth leading cause of cancer death worldwide [1]. Persistent hepatitis B virus (HBV) or hepatitis C virus (HCV) infection, alcohol abuse, and nonalcoholic fatty liver disease (NAFLD) have already been identified as the primary risk factors for the development of HCC. Despite the prevention of chronic HBV infection via universal HBV vaccination, which has drastically reduced the incidence of HCC, many unvaccinated people are still infected with HBV. Therefore, they are at risk for HCC development [2,3]. On the other hand, several studies have revealed that antiviral treatments using nucleos(t)ide analogs, which achieved sustained reduction but not the elimination of HBV-DNA, are closely associated with a decrease in HCC incidence [1,2,3,4]. Likewise, the incidence of HCC was also reduced in patients who underwent treatment with direct-acting antiviral agents (DAAs) against HCV and achieved a sustained viral response (SVR) [1,2,3,4]. However, the fact is that the number of NAFLD-related HCC patients is currently increasing worldwide [2,5].

Patients with HCC are generally characterized by having chronic liver damage including advanced liver fibrosis or liver cirrhosis as an underlying liver disease. The treatment algorithm for HCC thus considers the liver functional reserve as well as the size and number of tumors, vascular invasion, and distant metastasis [6,7,8]. These chronic liver diseases are frequently associated with several metabolic abnormalities including obesity and/or insulin resistance, which are widely known to play crucial roles in carcinogenesis. Such metabolic abnormalities are also attributed to the risk factors for HCC [9,10]. 

Some dietary factors including a high intake of sugar and saturated fat derived from red meat may be associated with an increased risk of HCC development. Other factors such as *n*-3 polyunsaturated fatty acid, coffee, and vitamin E may potentially have protective effects in at risk populations for HCC [11,12,13]. In addition, there is increasing evidence that supplementation with branched-chain amino acid results in an improvement of the prognosis of patients with HCC [13,14]. Therefore, nutritional intervention should be considered, especially in cirrhotic patients, for the prevention of HCC occurrence. 

Essential trace elements are well-recognized as dietary elements that are required in very minute quantities for the proper growth, development, and physiology of organisms. Impaired balance of several essential trace elements is involved in the development of a variety of cancers including colorectal, breast, and esophageal cancer as well as HCC [15,16,17,18]. While excessive levels of some essential trace elements such as iron (Fe) and copper (Cu) are associated with carcinogenesis, the development of various types of cancers have also been associated with deficient levels of other trace elements such as zinc (Zn) and selenium (Se). Therefore, the supplementation or depletion of these trace elements has been considered as potential therapeutic strategies for HCC.

In this review article, we primarily focus on the correlation between the dysregulation of the homeostasis of these three essential trace elements and the development of HCC and aim to summarize the current trends on the roles of these essential trace elements in the process of hepatocarcinogenesis. Moreover, we mention the efficacy as the novel treatments for HCC that target these trace elements.

## 2. Zinc

### 2.1. The Interaction between Zn Status and Carcinogenesis

Zn is an essential trace element that acts as a key constituent or cofactor of over 300 mammalian proteins. It plays crucial roles not only in stabilizing the structures of many proteins, but also participates in DNA synthesis. Zn is also involved in the activity of transcription factors, antioxidant defense, and DNA repair [19,20]. These processes are largely mediated by metallothioneins (MTs), which are cysteine-rich cytosolic proteins capable of binding to Zn and Cu [21]. 

**Figure 1:**
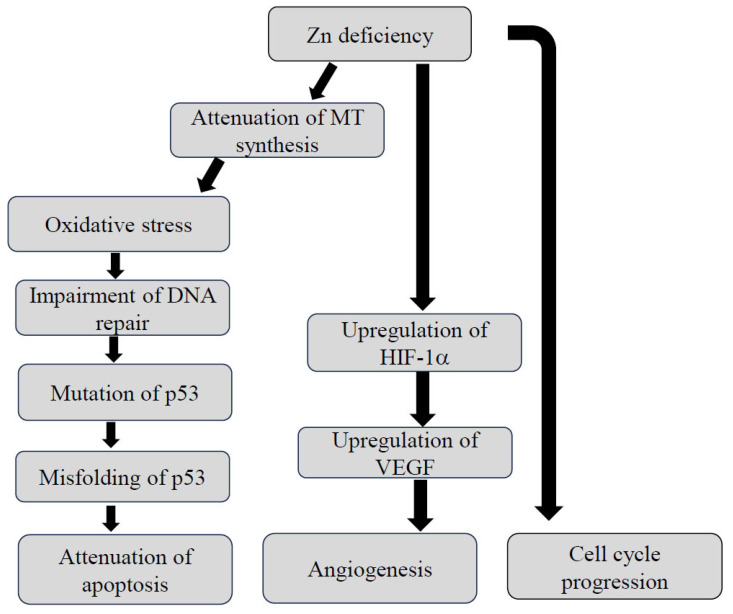
Putative mechanism by which dysregulation of Zn homeostasis causes carcinogenesis. HIF-1α: hypoxia-inducible factor-1α, MT: methallothioneine, VEGF: vascular endothelial growth factor, Zn: zinc.

The upregulation of MTs eventually leads to detoxification and protection against oxidative stress. The function of MTs is closely associated with Zn^2+^ redox status [22,23]. Major types of MT isoforms have been identified in mammals so far: MT1, MT2, MT3, and MT4 [24,25]. Some of these are involved in the process of carcinogenesis including the cell cycle arrest of tumor cells, facilitating the apoptosis of tumor cells as well as antioxidant effects on tumor cells. P53 is a zinc-binding transcription factor that can inhibit cell cycle progression and initiate apoptosis in response to DNA damage [26,27]. Therefore, Zn deficiency can result in DNA breaks and oxidative modification to DNA, which initiates carcinogenesis [28,29]. Figure 1 illustrates the process of carcinogenesis induced by Zn deficiency. Zn deficiency also promotes the cell cycle progression of the cells. Another study revealed that the overexpression of p53 was observed in human tumor cells under the condition of Zn deficiency. Zn deficiency induced the misfolding of p53 and subsequently formed non-functional p53 tetramers and aggregated misfolded p53 isomers in the cells. Both of these phenomena resulted in the attenuation of apoptosis, ultimately, carcinogenesis [30]. It is of interest that the administration of zinc chloride is likely to downregulate hypoxia-inducible factor 1α (HIF-1α) expression in human prostate cancer cells through the inhibition of vascular endothelial growth factor (VEGF) [31]. In addition, several microRNAs that potentially promote the development of esophageal cancer have been identified in Zn-deficient mice [32].

Zn manages to regulate the microenvironment created in the tumor. It is essential for the host’s defense against the initiation and progression of cancer as an immune-mediated property. Oral Zn intake was correlated with a reduced risk of cancers [33]. Zn plays crucial roles in the maintenance of the helper T lymphocyte1(Th1)/Th2 balance in the cancer immune microenvironment. The correct Th1/Th2 balance is required for the initiation of a proper immune response against tumor cells. Zn also facilitates the function of cytotoxic T lymphocytes (CTLs), which exert direct cytotoxic effects on tumor cells. Zn is necessary for the activation of natural killer (NK) cells, which are involved in the prevention of cancer development [34,35]. In addition, Zn shows favorable anticancer effects by attenuating the activity of signal transducer and activator of transcription 3 (STAT-3)-mediated in the development of Th17 lymphocytes [36]. 

### 2.2. Zn Status in Patients with HCC

Several studies on Zn status in patients with HCC have been reported to date [18,37,38]. A previous nested case–control study demonstrated that lower circulating Zn levels at baseline might predict the development of HCC [39]. However, serum Zn levels in liver cirrhosis patients with and without HCC were conflicting: several studies revealed that these levels were approximately equivalent between those patients with and without HCC [40,41], although it is well-established that zinc deficiency is frequently observed in liver cirrhosis patients [42]. Another report elucidated even lower Zn levels in those patients with HCC than in those without HCC [43]. Surprisingly, Fang et al. found no correlation between the serum Zn levels at the diagnosis of HCC and prognosis [44]. A 10-year follow-up study elucidated no correlation between the serum Zn level at baseline and the development of HCC in patients with HBV-related liver cirrhosis who were receiving nucleoside/nucleotide analogs or had received interferon therapy [45]. Dietary intake of Zn was also not associated with the risk of liver cancer in China [46]. Further epidemiological studies will be required to verify the correlation between zinc status and prognosis of other populations of HCC patients.

MTs also play essential roles in the prevention of HCC development. Previous studies have revealed that the expression of MT1G is mainly downregulated in HCC cell lines [47]. According to a recent study by Udai et al., the expressions of MT1G and MT1H were downregulated in human HCC tissues, and the expressions were dependent on the hepatic Zn contents [48]. In the Zn deficiency condition, the function of MT1G as an antioxidant action may be impaired, leading to the development of HCC.

It is of interest that HCC patients with lower serum Zn levels who underwent an initial hepatectomy showed unfavorable prognosis [49]. Likewise, early stage HCC patients with lower zinc levels who received curative local treatment showed worse overall survival [50]. In patients who achieve the eradication of HCV, lower Zn levels may predict the occurrence of HCC [51].

Several studies that have revealed that Zn contents are significantly lower in HCC tissues than those in surrounding liver tissues has accumulated thus far [18,37,38,48,52,53] because zinc regulates the proliferation, growth, and apoptosis of HCC cells. Cellular Zn homeostasis is largely mediated by two types of zinc transporters: the zinc transporter (ZnT) family and Zrt- and Irk-like protein (ZIP) family. The ZnT family acts as a Zn transporter that sequesters zinc among organelles or exports zinc from the cells, while ZIP transporters are required for zinc uptake from the extracellular fluid. Dysregulation of Zn transporters may be associated with the occurrence of several kinds of cancers [54]. In situ RT-PCR revealed that the expression of ZIP14 was markedly downregulated in HCC cells accompanied by a decrease in zinc content [55]. However, ZIP4 mRNA and protein were markedly elevated in HCC tissues than those in the non-cancerous surrounding tissues [56]. Gartmann et al. elucidated higher expressions of ZIP14 and ZIP4 in HCC tissues than in the surrounding non-tumor tissues. The severity of ZIP4 and 14 expressions in the HCC tissues was inversely correlated with overall survival [57].

It is of interest that five hub genes have been identified as key candidates that are potentially associated with the occurrence of HCC. Insulin-like growth factor-1 (IGF-1), one of the five hub genes linked with the development of HCC, was downregulated in the HCC tissues and enriched in cellular response in zinc ions. Therefore, a lower expression of IGF-1 may predict unfavorable prognosis of HCC patients [58].

### 2.3. The Effects of Zn Supplementation in HCC Cell Lines or Experimental HCC Models

Treatments with Zn compounds have shown favorable effects on HCC cell lines. Wang et al. demonstrated that a high concentration of zinc sulfate (ZnSO_4_) treatment (200 μM) resulted in the initiation of the apoptosis of HepG2 cells in 24 h [59]. Another study revealed that treatment with polaprezinc, a complex of zinc with L-carnosine, inhibited cell growth in HepG2 and Huh7 cells [60]. Recently, we reported that the administration of zinc acetate resulted in the induction of the apoptosis of Huh7 cells in 24 h [61].

Additional treatment of zinc to vitamin B17 (amygdalin) also acted as an anticancer agent through the remarkable apoptotic effect of HepG2 cells [62]. The zinc–curcumin complex significantly inhibited the growth of HepG2 cells in a dose-dependent manner [63]. Curcumin, which is a polyphenol compound isolated from turmeric rhizome, serves as a ligand of Zn and forms a complex with Zn [64]. Interestingly, the complex also enhanced the chemosensitivity to doxorubicin in the HepG2 cells [63].

The use of nanomaterials for the delivery of pharmaceutical or diagnostics agents has been considered as a potential cancer treatment. Zinc oxide nanoparticles displayed antitumor effects in both a cell line of HCC and a rat HCC model through the suppression of reactive oxygen species (ROS) generation [65].

### 2.4. The Preventive Effects of Zinc Supplementation on the Progression of HCC in the Clinical Trials 

Evidence that Zn supplementation shows preventive effects on the progression of HCC in patients with HCV-related chronic liver disease (CLD) is provided in Table 1. To the best of our knowledge, Matsuoka et al. were the first to demonstrate the clinical efficacy of Zn supplementation on the prevention of HCC development. The authors revealed that the serum Zn levels after the administration of polaprezinc were important for the prediction of HCC development in patients with CLD-C. Notably, CLD-C patients whose serum Zn levels were increased by the treatment with polaprezinc (Zn responder) had a lower cumulative incidence of HCC [66]. Later, the same group documented that additional treatment with polaprezinc to the hepatoprotective agents significantly inhibited the occurrence of HCC compared to the treatment with hepatoprotective agents alone [67]. Another study retrospectively verified the clinical efficacy of Zn supplementation in patients with CLD-C who achieved SVR. The cumulative incidence rate of HCC for 3 years was significantly lower in the group supplemented with zinc sulfate or zinc acetate (50–150 mg of Zn/day) compared to that in the group without Zn supplementation [68]. Taking these results into consideration, Zn supplementation may improve the immunological function for the prevention of HCC development. Cu deficiency should be noted when a high-dose of Zn supplementation is prolonged [69].

## 3. Copper

### 3.1. Cu Homeostasis

Cu is another essential trace element that is required for a wild range of physiological processes including maintaining DNA integrity, synthesizing essential metabolites, transporting oxygen to the mitochondrial respiratory chain, and involving redox reactions. It acts as a dynamic signaling metal and metalloallosteric regulator, participating in cell growth and proliferation, autophagy, and antioxidant defense [70,71]. There are four types of components involved in Cu homeostasis: (i) transporters that mediate Cu absorption (copper transporter receptor-1: CTR1), (ii) enzymes that initiate Cu ion efflux (ATP7A/B), (iii) biomolecules that sequester or store Cu (metallothionein), and (iv) Cu chaperones that deliver Cu to the organelles including copper chaperone for superoxide dismutase (CCS), superoxide dismutase-1 (SOD1), and antioxidant protein 1 (ATOX1) [70,71,72,73].

Extracellular Cu^2+^ is reduced to Cu^1+^ by the reductase protein family, STEAP proteins. Cu^+^ is delivered into the cells by way of CTR1, and its expression is mediated in a Cu-dependent manner [74]. The expression of CTR1 is downregulated under an excessive Cu state. The fraction of Cu^+^ is transported to cytosolic Cu chaperons such as CCS and SOD1 in order to scavenge free radicals [73,74]. Another chaperone of antioxidant protein 1 (ATOX1) can deliver Cu^+^ to copper-transporting ATPase 1 (ATP7A) and copper-transporting ATPase 2 (ATP7B), which are located in the trans-Golgi network and act as a major transporter for the export of cellular Cu. ATP7A transports Cu from the enterocytes to the blood, while ATP7B excretes Cu from the hepatocytes to the bile. These transporters also promote the synthesis of cuproproteins including ceruloplasmin and lysyl oxidase (LOX) for the removal of excessive Cu^+^ [73,74]. ATOX1 also delivers Cu into the nucleus, leading to the upregulation of a transcriptional activator of cyclin D [74]. The other chaperone, cytochrome c oxidase copper chaperone 17 (COX17), is responsible for the delivery of Cu^+^ to the mitochondrial intermembrane space to generate ATP [75,76]. 

Ceruloplasmin is a major carrier for Cu and binds to approximately 90% of serum Cu. Although ceruloplasmin synthesis and secretion are independent of serum Cu levels, Cu deficiency results in a decline in the stability and activity of ceruloplasmin [75,76]. Ceruloplasmin is also responsible for iron homeostasis as well as Cu metabolism. It facilitates the oxidization of iron ion from Fe^2+^ to Fe ^3+^ [77,78].

Excessive Cu^+^ accumulation in the cells leads to the production of reactive oxygen species (ROS), and subsequently the damage to the cells. In order to prevent Cu toxicity, excessive Cu is sequestered by MTs in a reaction probably mediated by glutathione (GSH). The synthesis of MTs is facilitated under the condition of oxidative stress [79].

### 3.2. The Relationship between Cu Status and Carcinogenesis

**Figure 2:**
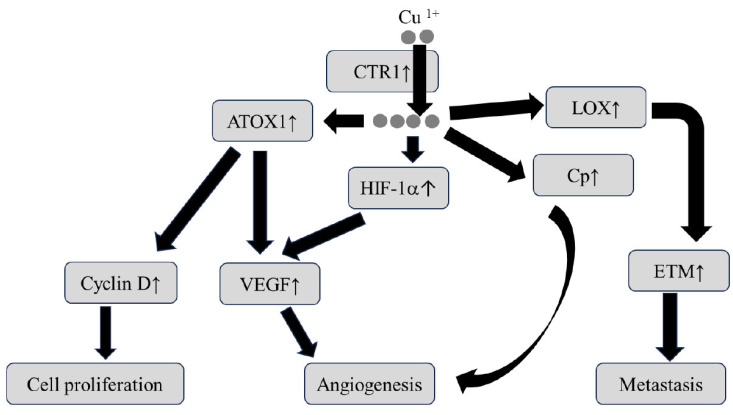
Putative mechanism by which the dysregulation of Cu homeostasis causes carcinogenesis. ATOX1: antioxidant protein 1, CTR1: copper transporter receptor 1, EMT: epithelial to mesenchymal transition, HIF-1α: hypoxia-inducible factor-1α, LOX: lysyl oxidase, VEGF: vascular endothelial growth factor. The arrow ↑ indicates activation.

Cu ions are considered harmful to the human body whether in excessive or deficient states. Cu-ion deficiency causes a decrease in the activities of multiple enzymes, leading to the impairment of energy level, impaired glucose tolerance, and dyslipidemia as well as bone marrow suppression. Cu deficiency affects the immune system [72,79]. In contrast, when Cu ions are in an excessive state, it enhances radical change and decreases the activity of proteins and enzymes, causing cellular injury by way of promoting oxidative stress, inflammation, and DNA damage [80]. Therefore, excessive Cu status is involved in malignant cell transformation as a central hub in the cell signaling pathway including cell proliferation, angiogenesis, and metastasis, as shown in Figure 2 [72,73,74]. 

Indeed, previous studies have documented that the expression of CTR1 is upregulated in various types of malignant tumors [74]. Cu uptake through CTR1 activates the mitogen activated protein kinase (MAPK) signal cascade, leading to the promotion of tumor growth [72,73,74]. The Cu chaperon ATOX1 is likely to serve as a modulator of tumor angiogenesis [73,74,81]. Cu regulates the hypoxia-inducible factor-1 (HIF-1) transcriptional complex and eventually mediates potent angiogenetic factors including vascular endothelial growth factor (VEGF) and ceruloplasmin [82]. Ceruloplasmin plays key roles not only in the transport of Cu, but also in the formation of novel blood vessels in cancer tissues. Indeed, the incidence of cancer development is associated with serum ceruloplasmin levels in several types of cancers [83,84], implying that serum ceruloplasmin levels may be a predictive hallmark for cancer development.

The family of Cu-dependent lysyl oxidase (LOX) and lysyl oxidase-like (LOXL) is involved in the metastasis of neoplastic cells [74,81]. The LOX family of enzymes mediates the crosslinking of collagen and elastin and promotes the maturation of the extracellular matrix. Cancer cells secrete LOX to remodel the extracellular matrix and create an environment more conductive to metastasis [74,81]. Previous studies have revealed that the expression of LOXL2 is upregulated in highly invasive cancers, and that LOXL2 ultimately leads to epithelial–mesenchymal transition by suppressing the expression of E-cadherin, which is a protein involved in tight junction [74,81,82].

It is of great interest that the Cu-ion concentration in tumor cells is strongly dependent on the expression of programed death-ligand 1 (PD-L1), which acts as an immune checkpoint inhibitor associated with cancer immune evasion. A recent study found the upregulation of PD-L1 gene expression in cancer cells by Cu supplementation [85]. The study also revealed that the administration of TEPA, a Cu chelator, initiated the degradation of PD-L1 and thereby inhibited tumor growth in a neuroblastoma xenograft mouse model, providing novel insights into the mechanism of tumor immune evasion.

Recently, a novel concept of “cuproptosis” has been established, which is recognized as copper-induced cell death, distinct from other cell death including apoptosis, ferroptosis, and necroptosis [86]. Cuproptosis may also be involved in the process of carcinogenesis. Potential therapeutic strategies that target cuproptosis have been developed for cancer patients [74,87]. 

### 3.3. The Relationship between Cu Metabolism and Chemoresistance in Cancer

Previous studies have revealed that the high affinity Cu transporter CTR1 mediated the cellular uptake of platinum-based chemotherapeutics such as cisplatin. Ishida et al. found that elevated levels of *Ctr1* mRNA were associated with sensitivity to platinum-based chemotherapy in human ovarian cancer [88]. In contrast, the low expression of CTR1 in patients with ovarian cancer showed a resistance to platinum-based chemotherapy [89]. However, the levels of the cellular uptake of the platinum-based drug were not correlated with the degree of CTR1 expression [88]. As an explanation to the result, Shanbhag et al. speculated that CTR1 mediated the uptake of cisplatin via an endocytic mechanism rather than direct transport across the lipid bilayer [90]. However, the detailed mechanism of platinum-based drug accumulation remains uncertain. 

In addition, Komatsu et al. found that the expression of ATP7B was upregulated in cisplatin-resistant prostate cancer cells [91]. The result indicated that ATP7B expression might also be associated with cisplatin-resistance in cancer cells. In fact, HCC patients with a high expression of ATP7B had an unfavorable prognosis [92].

### 3.4. Cu Status in Patients with HCC

Evidence that HCC patients have higher serum Cu levels than those with chronic liver disease has accumulated [93]. Cu accumulation is also more severe in HCC tissues compared to that in the surrounding liver parenchyma [18,52,53]. Therefore, it is conceivable that the incidence of HCC is significantly higher in patients with Wilson’s disease, which is well-recognized as a Cu overload [94,95].

An increase in the ratio of serum Cu to Zn levels (Cu/Zn) may be a predictive marker for the development of HCC [39,44,96], as it is in other malignant tumors [97,98]. The Cu/Zn ratio may also indicate the prognosis of HCC patients [44,99]. However, dietary Cu intake is not associated with the risk of liver cancer [46].

According to a previous study by Ebara et al., no significant differences in MT levels were found between HCC and the surrounding liver parenchyma [100]. The levels of MTs in HCC tissues were independent of tumor size. It is noteworthy that the fraction of Cu-MT was significantly increased in HCC compared to the fraction in the surrounding liver parenchyma. 

Ceruloplasmin is also associated with the occurrence of HCC. A previous study documented that serum ceruloplasmin levels were higher in patients with HCC compared to those in patients with liver cirrhosis [101]. Similar results were obtained between patients with HCV-induced alcoholic liver cirrhosis with and without HCC [102]. It is of interest that the core-fucosylation changes of ceruloplasmin were useful to distinguish the alcohol-related HCC from alcoholic liver cirrhosis. The changes were not valid between HBV-or HCV-related HCC and HBV-or HCV-related liver cirrhosis [103], although the reason remains unclear. In a recent in vitro study, the deletion of ceruloplasmin resulted in the facilitation of ferroptosis [104], which is programmed cell death dependent on iron ions and different from apoptosis, necrosis, and autophagy, in HCC cells [105].

The association between the expression of Cu-transporter proteins and other clinical parameters have been verified in HCC patients. Porcu et al. documented that the expression of CTR1 protein was more upregulated in nonalcoholic steatohepatitis (NASH)-related HCC tissues compared to that in the underlying NASH tissues, and that its expression was paralleled with MYC expression in those patients. The result might suggest that MYC binds to a specific lesion of the CTR1 promotor and then mediates the transcription [91]. On the other hand, some HCC patients displayed ATP7B expression in the bile duct epithelial cells as well as the tumor cells. The expression of ATP7B may predict cisplatin-resistance in those patients [92]. Another report revealed that HCC patients with a high expression of COX17 displayed more favorable prognosis. Interestingly, the COX 17 expression was negatively correlated with CD274, which referred to PD-L1, in such patients [106]. 

HIF-1α is also involved in the development of HCC [107]. Our previous study revealed that the serum HIF-1α levels were significantly correlated with the serum Cu levels in HCC patients [108]. We speculate that Cu accumulation resulted in the upregulation of HIF-1α expression, promoting the transcription of genes responsible for angiogenesis in those patients.

### 3.5. Therapeutic Strategies Which Target Cu in HCC Cell Lines or Experimental HCC Models

Targeting Cu ions could become a promising candidate for cancer treatment. Several Cu chelators and Cu ionophores are well-established as the main therapeutic strategies for targeting Cu in cancers [72,73,74,81,82]. Cu chelators reduce the bioavailability of copper by binding to Cu, leading to the inhibition of carcinogenesis such as angiogenesis, tumor growth, and metastasis. The antitumor effects of some Cu-chelating agents including trientine, tetrathiomolybdate (TTM), and d-penicillamine have been fully recognized in human studies as well as animal models [72,73,81,82]. Trientine and TTM are largely selected as Cu chelators in HCC cell lines or experimental HCC models.

Sone et al. demonstrated that the long-term administration of trientine suppressed the incidence of HCC in Long–Evans Cinnamon (LEC) rats, which have been developed as an experimental animal mode of Wilson’s disease [109]. In a murine HCC model, the administration of trientine resulted in the inhibition of angiogenesis and the induction of apoptosis in the tumor cells [110]. The antiangiogenic action by trientine may derive from the inhibition of interleukin-8 (IL-8) production from HCC cells [111]. Another Cu chelator, TTM, can also alleviate tumorigenic properties in HCC cell lines in a dose-dependent manner [112]. The same authors revealed that TTM-induced Cu deficiency caused the attenuation of glycolysis under the hypoxic condition, leading to a decrease in glucose utilization and lactate excretion [112].

In contrast, Cu ionophores can raise the intracellular Cu levels and exert antitumor effects through the induction of ROS, suppression of proteosome activity, and ultimately, apoptosis in cancer cells. Chloroquinol, disulfiram (DSF), and elesclomol are primarily utilized as Cu ionophores that possess antitumor properties. 

DSF is an aldehyde dehydrogenase inhibitor that is originally used as a therapeutic agent for alcoholism [74]. DSF turns out to be highly toxic in cancer cells in a Cu-dependent manner [113]. Cu ions react with DSF to form a complex of diethyldithiocarbamate-copper. The complex selectively acts on the tumor cells. A previous in vitro study provided evidence that DSF combined with Cu inhibited the proliferation, migration, and invasion of HCC cells through the suppression of nuclear factor-κB (NF-κB) and transforming growth factor-β (TGF-β) signaling [114]. In addition, the combined treatment of DSF/Cu with sorafenib, which is a multi-tyrosine kinase inhibitor and is well-recognized as a molecular target medicine for advanced HCC [115], had synergistic effects on HCC cells via the inhibition of nuclear factor erythroid2-related factor 2 (NRF2) and MAPK activity [116]. Another study revealed that DSF/Cu upregulated PD-L1 expression in the murine HCC model through the inhibition of poly(ADP-ribose) polymerase 1 (PARP1) activity, the enhancement of glycogen synthase kinase-3β (GSK-3β) phosphorylation at Ser9, and the consequent inhibition of T cell infiltration [117].

Elesclomol is a novel Cu ionophore that directly transports Cu ions to the mitochondria inside cells [73,74]. Currently, findings that elesclomol can induce cuproptosis in HCC cell lines in a dose-dependent manner have been reported [118,119]. 

Recently, CD 147, which is a type I transmembrane glycoprotein, has been identified as a novel molecular target of Cu in the treatment for HCC [120]. CD 147 is highly expressed in a various type of cancers and plays an important role in the signaling receptor for extracellular Cu^2+^ in cancer patients [121]. 

Ionic ^64^CuCl_2_ is considered to be a potentially therapeutic radiopharmaceutical in tumors that express high levels of human copper transporter 1 (hctr1) [122]. Therefore, ^64^CuCl_2_ may become a promising radionuclide therapy for HCC [123] because the upregulation of the hctr1 gene is frequently observed in HCC cells. 

The use of copper oxide nanoparticles (CuONPs) was applied as a therapeutic agent for HCC in one in vitro study. Siddiqui et al. revealed the antitumor effects of CuONPs in Hep G2 cells via the upregulation of caspase-3 gene expression [124].

### 3.6. Therapeutic Strategies Which Target Cu in Patients with HCC

No clinical trials on the efficacy of Cu chelators or Cu ionophores in HCC patients have been conducted so far, although several clinical trials (phase I or phase II) on the efficacy of a Cu chelator, TTM, have already been completed in other types of cancer patients [125,126]. It is noteworthy that the serum ceruloplasmin levels were monitored during the trial as a surrogate marker for total body copper [125]. 

D-penicillamine is widely utilized as a therapeutic agent for Wilson’s disease to improve Cu deposition in the liver. Surprisingly, patients who are treated with d-penicillamine have an increased risk of HCC development because the administration of d-penicillamine often causes iron accumulation and synergistic radical formation in the liver [127].

Many candidates have been proposed as therapeutic agents that target Cu in HCC patients. Some of them may become complementary therapeutic strategies for HCC to enhance the efficacy of the molecular targeting agents. Clinical application of these therapeutic agents is urgently required.

## 4. Selenium

### 4.1. The Relationship between Se Status and Carcinogenesis

Selenium (Se) is also an essential trace element that is required for human health. It serves as antioxidant and detoxication actions [128,129]. The fact is that the optimal amount of Se necessary for cellular function is in a narrow range. In the Se deficient state, various types of symptoms or disorders occur due to the decreased activity of Se-containing enzymes. An excessive Se state, on the other hand, can be toxic to bodies.

**Figure 3:**
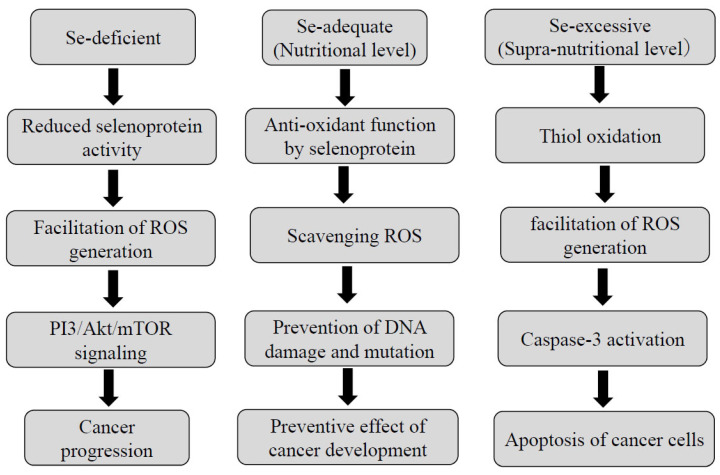
The roles of selenium depending on its concentration.

The involvement of Se in carcinogenesis is primarily dependent on its concentration, as illustrated in Figure 3. In the Se-deficient state, ROS generation is facilitated due to decreased activities of selenoproteins including glutathione peroxidases (GPxs) and thioredoxin reductases (TrxRs) [130,131,132]. A moderate degree of ROS leads to cancer progression by way of PI3/Akt/mTOR signaling [133]. Se deprivation can also upregulate the expression of cell cycle-related genes including c-Myc and cyclin C [134].

At the nutritional Se level, which is defined as the amount sufficient to saturate selenoproteins [130,131,132,133], these selenoproteins serve as scavengers of ROS and prevent DNA damage and mutation. Thus, Se plays an antioxidant role in the process of carcinogenesis at a nutritional Se level. 

Whereas at a supra-nutritional Se level, which indicates a nontoxic dose greater than that required to support the maximal expression of the selenoenzymes [130], Se serves as a pro-oxidant agent in cancer cells [130,131,132,133,135,136,137]. A supra-nutritional Se level causes thiol oxidation, a high degree of ROS, and finally alleviates cancer progression through caspase-3-induced apoptosis of the tumor cells. A supra-nutritional Se level by a long-term treatment with sodium selenite, one of the inorganic forms of Se, can cause DNA damage in HCC cells [138]. It is of particular interest that selenite-induced DNA damage was associated with the induction of p53 in cervical cancer cells [139]. Such a Se level may also affect the late stage of carcinogenesis like apoptosis. Moreover, the supra-nutritional Se level by treatment with sodium selenite can cause G2/M cell cycle arrest in colon cancer cells [140]. Corcoran et al. demonstrated that the supra-nutritional dose of sodium selenite significantly retarded the growth of primary prostatic cancer and the development of lymph node metastases in murine, being accompanied by the inhibition of angiogenesis [141].

Se regulates both the innate and adaptive immune systems in cancer patients [130]. Sodium selenite supplementation displays antitumor effects by promoting the recruitment of CTLs and M1 polarization of macrophages in the tumor microenvironment [142,143]. A Se-containing complex enhanced the activity of NK cells against prostatic cancer cells through tumor necrosis factor-related apoptosis-inducing ligand (TRAIL) signaling [144]. Selenium nanoparticles (Se NPs) have the possibility of stimulating the maturation of dentritic cells with antigen-presenting function through regulating selenoproteins [145].

### 4.2. Interaction of Se with Other Essential Trace Elements 

Combined supplementation with Se and Zn has largely been conducted in patients with prostatic cancer [146]. However, it should be noted that Se supplementation may affect the Zn status and consequent dysregulation of MT synthesis [132]. Therefore, Se supplementation may affect Zn homeostasis in patients with prostatic cancer. 

It is of interest that a high dose of sodium selenite caused ferroptosis, which is a new type of cell death that is different from apoptosis, necrosis, and autophagy [147] in ovarian cancer cells [148]. Excessive Se supplementation generated a high degree of ROS and dysregulated GPx4 activities, which play an important role in the prevention of lipid peroxidation [149]. Therefore, a decrease in GPx4 activity results in lipid peroxide accumulation, iron overload, and ultimately, ferroptosis [150]. This is why a high dose of Se supplementation affects the iron status.

### 4.3. Se and Chemosensitivity in Cancer

Liu et al. investigated the correlation between trace elements and the sensitivity of cytotoxic anticancer agents in HCC patients. It is noteworthy that the carboplatin sensitivity was inversely corelated with the Se level in the cancer tissues [151]. In addition, evidence that Se nanoparticles (SeNPs) potentially raise the chemosensitivity of anticancer agents has been provided. For example, SeNPs initiated the chemosensitivity of fluorouracil nanoparticles in the breast and colon cancer cell lines [152]. Another study documented that the Se–sorafenib nanocomplex could bypass the chemoresistance in glioblastoma cells [153]. It is of particular interest that this Se–sorafenib nanocomplex induced the apoptosis of cancer cells via Ca^2+^-dependent endoplasmic reticulum stress.

### 4.4. Se Status in Patients with HCC

It is well-recognized that serum Se levels are gradually decreased as the severity of hepatic fibrosis becomes more severe [154]. However, it remains controversial whether the serum Se levels are lower in patients with HCC compared to those in patients with liver cirrhosis [155,156]. A previous large prospective cohort study revealed that higher serum selenoprotein P as well as Se levels were associated with a lower risk of HCC occurrence [157]. Likewise, several meta-analysis studies elucidated an inverse correlation between the serum Se levels and the risk of HCC in human populations [158,159]. 

The correlation between dietary Se intake and the incidence of HCC was also explored. Unexpectedly, the amount of dietary Se intake was not associated with the incidence of HCC [46,159]. 

The association of serum Se levels with tumor sizes were also investigated in HCC patients. Rotor-Udilova et al. documented that the serum Se levels were inversely correlated with tumor sizes in HCC patients, although the correlation was limited within diameters less than 3 cm [160]. The authors also confirmed inverse correlations between the serum Se levels and VEGF or IL-8 levels in those patients. In addition, the Se content in HCC tissue declined in proportion to the malignant grade. The Se content in the tumor tissue was significantly lower compared to that in the liver of the normal control [161].

Several studies have elucidated the clinical characteristics of the expression of selenoproteins in human HCC tissues. HCC patients with a higher expression of GPx4 significantly exhibited more a favorable prognosis than those with a lower expression of GPx4 because GPx4 inhibited the development of HCC by way of the regulation of angiogenesis and the modulation of immune-mediated cells [162]. The authors demonstrated that regulatory T cells and NK cells were recruited more, but the grade of the γδT cells and infiltration of the activated dendritic cells were decreased in tumors with high GPx4 expression. A shift in macrophage distribution was also observed from M2 to M1 in such HCC tissues. In contrast, a low expression of GPx4 in the HCC tissues indicated an unfavorable overall survival rate [163]. Another study documented that the expression of GPx4 was stronger in HCC tissues with high-grade malignancy than that in those with low- or moderate-grade malignancy, which is shown in the conflicting results described above [164]. 

GPx1 participates in the neutralization of hydrogen peroxide (H_2_O_2_) and organic hydroperoxides by preventing damage to mitochondrial DNA and the protection of cells from free radicals [165]. Activated GPx1 can protect cancer cells from ROS and anticancer agents. On the other hand, selenium-binding protein-1 (SBP-1) is a selenium-containing protein that transports Se [166]. SBP-1 is extensively expressed in normal liver tissues, although its expression is weak in HCC tissues [167]. Decreased SBP-1 expression resulted in the macrovascular invasion of HCC by way of increasing the GPx1 activity and diminishing HIF-1α expression [168] or the upregulation of C-X-C motif chemokine receptor 4 (CXCR4) [169] in the HCC tissues.

The relationship between the genetic polymorphism of GPx1 and cancer development has also been explored in various types of cancers. Sutton et al. revealed that the GPx1 polymorphism was associated with the occurrence of HCC in patients with alcoholic liver cirrhosis. The authors identified two pro-GPx1 alleles that indicated the low incidence of HCC in such patients [170].

Thioredoxin reductase (TrxR1) is an important selenocysteine (Sec)-containing antioxidant enzyme that is involved in the reduction of oxidized thioredoxin-1 (Trx1) [165]. It is well-known that TrxR1 is upregulated in many malignant diseases, and that it can promote tumor growth [171]. Indeed, the expression of TrxR1 protein was much higher in human HCC tissues compared to that in non-tumorous lesions. The severity of TrxR1 expression in the HCC tissues was associated with tumor stage. Moreover, HCC patients with high TrxR1 expression displayed unfavorable prognosis [172,173]. Likewise, higher serum TrxR1 levels in patients with HCC may predict poor prognosis including the recurrence of HCC [174]. Recently, Hua et al. demonstrated that TrxR1 was directly targeted by miR-125-5p in those patients [175].

### 4.5. Therapeutic Strategies Which Target Se in HCC Cell Lines or Experimental HCC Models 

The administration of the inorganic form of Se, sodium selenite, has been used as a therapeutic strategy for HCC in in vitro and in vivo studies. It is noteworthy that the antitumor effect of sodium selenite, which belongs to an inorganic selenium compound, is dependent on its concentration. The administration of sodium selenite eventually causes the apoptosis of Hep G2 cells in a dose-dependent manner ranging from 10 μM to 50 μM [176]. Another study revealed that treatment with 50 nM of sodium selenite increased GPx4 expression and decreased VEGF expression as well as tumor growth in HCC cells [160]. The antitumor effects of selenium sulfide (SeS_2_), which is another inorganic type of selenium compound and was originally used as a therapeutic agent for seborrheic dermatitis [177], were also confirmed in HCC cell lines [178]. 

Selenium methylselenocysteine (SeMSC) is an organic form of selenium compound that is also used as a therapeutic strategy for HCC. Treatment with SeMSC had a remarkable protective effect on HepG2 cells including decreases in malondialdehyde (MDA) concentration and GPx activity in the nanomolar to micromolar range [179]. The efficacy of selenium-enriched malt (SEM), another type of organic Se compound, on rat HCC was also confirmed. The treatment with SEM improved the mortality and reduced the number of HCC nodules by inhibiting the expression of VEGF and protein kinase C-α in the tumor tissues [180]. The inhibitory effects of SEM on rat HCC proved to be stronger than those of sodium selenite. It is of interest that the Se-enriched Grifola frondosa polysaccharide (Se-GP11) enhanced the antitumor effects of 5-fluorouracil (5-Fu) on Heps-bearing mice through an increase in SOD activities and a decrease in MDA levels [181]. 

Se-nanoparticles (SeNPs) may be a novel therapeutic strategy to overcome HCC in a drug delivery system because it possesses lower toxicity and higher bioavailability compared to organic and inorganic Se compounds. It serves as a mRNA-based nanocarrier [182]. Singh et al. developed the method for the delivery of *Fluc*-mRNA to HepG2 cells by functionalized SeNPs [183]. *Fluc*-mRNA was selected to confirm the transfection efficiencies in HCC cells. In addition, galactose-modified Se nanoparticles loaded with doxorubicin were developed to improve the antitumor efficacy of doxorubicin against HCC [184]. Se nanoparticles also had the ability to overcome sorafenib resistance in a rat HCC model by modulating apoptosis and mTOR/NF-κB signaling [185]. 

Recently, the efficacy of novel TrxR1 inhibitors including butaselen and piperlongumine have been verified in HCC cells or murine HCC models [173,186,187,188]. There may be a possibility for these TrxR1 inhibitors to become a promising therapeutic strategy for HCC. 

### 4.6. Therapeutic Strategies Which Targeting Se in Clinical Studies

Several clinical trials on the efficacy of treatment with selenium compounds alone or combined treatment of a Se compound with antioxidants have been performed in breast, colorectal, and prostate cancer [146,165], although its efficacy has not been verified in patients with HCC. We need to determine which type of Se compound is the most effective, or how much and how long the Se compound is administrated in such patients. The administration of a Se compound may be effective in light of an increase in the chemosensitivity of anticancer agents or immune check point inhibitors in HCC patients. Further clinical trials on the efficacy of Se compounds should be considered in these patients.

## 5. Conclusions

The efficacy of Zn supplementation for the prevention of HCC development has been confirmed in several clinical trials, although the efficacy of Cu depletion and Se supplementation has not. The optimal dose of Zn and the most effective type of Zn compound should be immediately determined for the prevention of HCC development. Cu chelators and Cu ionophores are widely recognized as potential therapeutic agents for HCC. Likewise, inorganic and organic Se compounds also seem to be promising in such patients. Moreover, numerous candidates that serve as carrier proteins for Zn and Cu or Se-containing enzymes have been identified as potential therapeutic strategies in HCC cell lines and experimental HCC models. The development of Zn-, Cu-, and Se-nanoparticles may enhance the antitumor effects through the improvement of the drug delivery system in HCC patients. These therapeutic agents should be clinically applied to these patients in the near future.

## Figures and Tables

**Table 1:** Clinical efficacy of Zn supplementation for the prevention of HCC development.

Reference	Study Design	Assigned Patients	Formulation	Dosage and Duration	Outcomes
Matsuoka et al. (2009) [66]	Prospective study (Zn responders vs. Zn non-responders)	HCV-related CLD (n = 32)	Polaprezinc	150 mg, 5 years	Lower incidence of HCC in Zn responders
Matsumura et al. (2012) [67]	Randomized control study (Zn group vs. untreated group)	HCV-related CLD (n = 62)	Polaprezinc	150 mg, 10 years	Lower incidence of HCC in Zn group
Hosui et al. (2021) [68]	Retrospective study (Zn group vs. untreated group)	HCV-related CLD who achieved SVR (n = 599)	Zinc sulfate or zinc acetate	Zinc 50–150 mg, 3 years	Lower incidence of HCC in Zn group

CLD: chronic liver disease; HCC: hepatocellular carcinoma; HCV: hepatitis C virus; SVR: sustained virological response; Zn: zinc.

## Data Availability

Not applicable.

## Abbreviations

ATOX1	Antioxidant protein 1
ATP7A/B	Copper-transporting ATPase 1/2
CLD	Chronic liver disease
COX17	Cytochrome C oxidase 17
CTL	Cytotoxic T lymphocytes
CTR1	Copper transporter receptor 1
Cu	Copper
DSF	Disulfiram
EMT	Epithelial mesenchymal transition
GPx	Glutathione peroxidase
HBV	Hepatitis B virus
HCC	Hepatocellular carcinoma
HCV	Hepatitis C virus
HIF-1α	Hypoxia-inducible factor-1α
LOX	Lysyl oxidase
LOXL	Lysyl oxidase-like
MAPK	Mitogen-activated protein kinase
MT	Methallothioneine
NK	Natural killer
PD-L1	Programed death-ligand 1
ROS	Reactive oxygen species
Se	Selenium
SeNP	Selenium nanoparticle
SVR	Sustained viral response
TrxR	Thioredoxin reductase
TTM	Tetrathiomolybdate
VEGF	Vascular endothelial growth factor
ZIP	Zrt- and Irk-like protein
Zn	Zinc

## References

[1] Yang J.D., Hainaut P., Gores G.J., Amadou A., Plymoth A., Roberts L.R. (2019). A global view of hepatocellular carcinoma: Trends, risk, prevention and management. Nat. Rev. Gastroenterol. Hepatol..

[2] Villanueva A. (2019). Hepatocellular carcinoma. N. Engl. J. Med..

[3] McGlynn K.A., Petrick J.L., El-Serag H.B. (2021). Epidemiology of hepatocellular carcinoma. Hepatology.

[4] Kulik L., El-Serag H.B. (2019). Epidemiology and management of hepatocellular carcinoma. Gastroenterology.

[5] Shah P.A., Patil R., Harrison S.A. (2023). NAFLD-related hepatocellular carcinoma: The growing challenge. Hepatology.

[6] European Association for the Study of the Liver (2018). EASL Clinical practice guidelines: Management of hepatocellular carcinoma. J. Hepatol..

[7] Marrero J.A., Kulik L.M., Sirlin C.B., Zhu A.X., Finn R.S., Abecassis M.M., Roberts L.R., Heimbach J.K. (2018). Diagnosis, staging, and management of hepatocellular carcinoma: 2018 practice guideline by the American Association for the Study of the Liver Diseases. Hepatology.

[8] Hasegawa K., Takemura N., Yamashita T., Watadani T., Kaibori M., Kubo S., Shimada M., Nagano H., Hatano E., Aikata H. (2023). Clinical practice guideline for hepatocellular carcinoma: The Japan Society of Hepatology 2021 version (5th JSH-HCC guidelines). Hepatol. Res..

[9] Siddique A., Kowdley K.V. (2011). Insulin resistance and other metabolic risk factors in the pathogenesis of hepatocellular carcinoma. Clin. Liver Dis..

[10] Chettouh H., Lequoy M., Fartoux L., Vigouroux C., Desbois-Mouthon C. (2015). Hyperinsulinemia and insulin signaling in the pathogenesis and the clinical course of hepatocellular carcinoma. Liver Int..

[11] Mandair D.S., Rossi R.E., Pericleous M., Whyand T., Caplin M. (2014). The impact of diet and nutrition in the prevention and progression of hepatocellular carcinoma. Expert. Rev. Gastroenterol. Hepatol..

[12] Koumbi L. (2017). Dietary factors can protect against liver cancer development. World J. Hepatol..

[13] Ruiz-Margáin A., Román-Calleja B.M., Moreno-Guillén P., González-Regueiro J.A., Kúsulas-Delint D., Campos-Murguía A., Flores-García N.C., Macías-Rodríguez R.U. (2021). Nutrition therapy for hepatocellular carcinoma. World J. Oncol..

[14] Yang W.-S., Zeng X.-F., Liu Z.-N., Zhao Q.-H., Tan Y.-T., Gao J., Li H.-L., Xiang Y.-B. (2020). Diet and liver cancer risk: A narrative review of epidemiological evidence. Br. J. Nutr..

[15] Juloski J.T., Rakic A., Ćuk V.V., Ćuk V.M., Stefanović S., Nikolić D., Janković S., Trbovich A.M., De Luka S.R. (2020). Colorectal cancer and trace elements alteration. J. Trace Elem. Med. Biol..

[16] Iqbal S., Ali I. (2022). Dietary trace element intake and risk of breast cancer: A mini review. Biol. Trace Elem. Res..

[17] Yang X., Tang Z., Li J., Jiang J. (2022). Esophagus cancer and essential trace elements. Front. Public Health.

[18] Gurusamy K. (2007). Trace element concentration in primary liver cancers-a systemic review. Biol. Trace Elem. Res..

[19] Vallee B.L., Falchuk K.H. (1993). The biochemical basis of zinc physiology. Physiol. Res..

[20] Prasad A.S. (2014). Zinc is an antioxidant and anti-inflammatory agent: Its role in human health. Front. Nutr..

[21] Lubna S., Ahmad R. (2023). Clinical and biochemical understanding of zinc interaction during liver diseases: A paradigm shift. J. Trace Elem. Med. Biol..

[22] Vasak M. (2005). Advances in metallothionein structure and functions. J. Trace Elem. Med. Biol..

[23] Bell S.G., Valee B.L. (2009). The metallothionein/thionein system: An oxidoreductive metabolic zinc link. Chemobiochem.

[24] Krizkova S., Ryvolova M., Hrabeta J., Adam V., Stiborova M., Eckschlager T., Kizek R. (2012). Metallothioneins and zinc in cancer diagnosis and therapy. Drug Metab. Rev..

[25] Moleirinho A., Carneiro J., Matthiesen R., Silva R.M., Amorim A., Azevedo L. (2011). Gains, losses and changes of function after gene duplication: Study of the metallothionein family. PLoS ONE.

[26] Si M., Lang J. (2018). The roles of metallothioneins in carcinogenesis. J. Hematol. Oncol..

[27] Franklin R.B., Costello L.C. (2009). The important roles of apoptotic effects of zinc in the development of cancers. J. Cell Biochem..

[28] Ho E. (2004). Zinc deficiency, DNA damage and cancer risk. J. Nutr. Biochem..

[29] Chasapis C.T., Loutsidou A.C., Spiliopoulou C.A., Stefanidou M.E. (2012). Zinc and human health: An update. Arch. Toxicol..

[30] Zang Y., Tian Y., Zhang H., Xu B., Chen H. (2021). Potential pathways of zinc deficiency-promoted tumorigenesis. Biomed. Pharmacother..

[31] Nardinocchi L., Pantisano V., Puca R., Porru M., Aiello A., Grasselli A., Leonetti C., Safran M., Rechavi G., Givol D. (2010). Zinc downregulates HIF-1 and inhibited its activity in tumor cells in vitro and in vivo. PLoS ONE.

[32] Alder H., Taccioli C., Chen H., Jiang Y., Smalley K.J., Fadda P., Ozer H.G., Huebner K., Farber J.L., Croce C.M. (2012). Dysregulation of miR-31 and miR-21 induced by zinc-deficiency promotes esophageal cancer. Carcinogenesis.

[33] Janakiram N.B., Mohammed A., Madka V., Rao C.V. (2016). Prevention and treatment of cancers by immune modulating nutrients. Mol. Nutr. Food Res..

[34] Prasad A.S., Beck F.W.J., Snell D.C., Kucuk O. (2009). Zinc in cancer prevention. Nutr. Cancer.

[35] John E., Laskow T.C., Buchser W.J., Pitt B.R., Basse P.H., Butterfield L.H., Kalinski P., Lotze M.T. (2010). Zinc in innate and adaptive tumor immunity. J. Trans. Med..

[36] Skarajnowska D., Bobrowska-Korczak B. (2019). Role of zinc in immune system and anti-cancer defense mechanisms. Nutrients.

[37] Stamoulis I., Kouraklis G., Theocharis S. (2007). Zin and the liver: An active interaction. Dig. Dis. Sci..

[38] Grüngreiff K., Reinhold D., Wedemeyer H. (2016). The role of zinc in liver cirrhosis. Ann. Hepatol..

[39] Stepien M., Hughes D.J., Hybsier S., Bamia C., Tjønneland A., Overvad K., Affret A., His M., Boutron-Ruault M.C., Katzke V. (2017). Circulating copper and zinc levels and risk of hepatobiliary cancers in Europeans. Br. J. Cancer.

[40] Nagasue N., Kohno H., Chang Y.C., Nakamura T. (1989). Iron, copper and zinc levels in serum and cirrhotic liver of patients with and without hepatocellular carcinoma. Oncology.

[41] Moriyama M., Matsumura H., Fukushima A., Ohkido K., Arakawa Y., Nirei K., Yamagami H., Kaneko M., Tanaka N., Arakawa Y. (2006). Clinical significance of evaluation of serum zinc concentrations in C-viral chronic liver disease. Dig. Dis. Sci..

[42] Himoto T., Masaki T. (2020). Current trends of essential trace elements in patients with chronic liver diseases. Nutrients.

[43] Shigefuku R., Iwasa M., Katayama K., Eguchi A., Kawaguchi T., Shiraishi K., Ito T., Suzuki K., Koreeda C., Ohtake T. (2019). Hypozincemia is associated with human hepatocarcinogenesis in hepatitis C virus-related liver cirrhosis. Hepatol. Res..

[44] Fang A., Chen P., Wang X., Liu Z., Zhang D., Luo Y., Liao G., Long J., Zhong R., Zhou Z. (2019). Serum copper and zinc levels at diagnosis and hepatocellular carcinoma survival in the Guangdong Liver Cancer Cohort. Int. J. Cancer.

[45] Wang S., Fan X., Gao Y., Zuo Y., Hong M., Xu Y. (2023). The relationship between zinc deficiency and hepatocellular carcinoma associated with hepatitis B liver cirrhosis: A 10-year follow-up study. Biol. Trace Elem. Res..

[46] Ma X., Yang Y., Li H.L., Zheng W., Gao J., Zhang W., Yang G., Shu X.O., Xiang Y.B. (2017). Dietary trace element intake and liver cancer risk: Results from two population-based cohorts in China. Int. J. Cancer.

[47] Wang Y., Wang G., Tan X., Ke K., Zhao B., Cheng N., Dang Y., Liao N., Wang F., Zheng X. (2019). MT1G serves as a tumor suppressor in hepatocellular carcinoma by interacting with p53. Oncogenesis.

[48] Udali S., De Santis D., Mazzi F., Moruzzi S., Ruzzenente A., Castagna A., Pattini P., Beschin G., Franceschi A., Guglielmi A. (2021). Trace elements status and metallothioneins DNA methylation influence human hepatocellular carcinoma survival rate. Front. Oncol..

[49] Harimoto N., Araki K., Muranushi R., Hoshino K., Yamanaka T., Hagiwara K., Ishii N., Tsukagoshi M., Watanabe A., Shirabe K. (2022). Significance of zinc deficiency in patients with hepatocellular carcinoma undergoing hepatic resection. Hepatol. Res..

[50] Hiraoka A., Nagamatsu K., Izumoto H., Adachi T., Yoshino T., Tsuruta M., Aibiki T., Okudaira T., Yamago H., Iwasaki R. (2020). Zinc deficiency as an independent prognostic factor for patients with early hepatocellular carcinoma due to hepatitis virus. Hepatol. Res..

[51] Ozeki I., Nakajima T., Suii H., Tatsumi R., Yamaguchi M., Arakawa T., Kuwata Y. (2020). Predictors of hepatocellular carcinoma after hepatitis C virus eradication following direct-acting antiviral treatment: Relationship with serum zinc. J. Clin. Biochem. Nutr..

[52] Ebara M., Fukuda H., Hatano R., Yoshikawa M., Sugiura N., Saisho H., Kondo F., Yukawa M. (2003). Metal contents in the liver of patients with chronic liver disease caused by hepatitis C virus. Reference to hepatocellular carcinoma. Oncology.

[53] Tashiro H., Kawamoto T., Okubo T., Koide O. (2003). Variation in the distribution of trace elements in hepatoma. Biol. Trace Elem. Res..

[54] Pan Z., Choi S., Ouadid-Ahidouch H., Yang J.M., Beattie J.H., Korichneva I. (2017). Zinc transporters and dysregulated channels in cancers. Front. Biosci..

[55] Franklin R.B., Levy B.A., Zou J., Hanna N., Desouki M.M., Bagasra O., Johnson L.A., Costello L.C. (2012). ZIP 14 zinc transporter down-regulation and zinc depletion in the development and progression of hepatocellular cancer. J. Gastrointest. Cancer.

[56] Weaver B.P., Zhang Y., Hiscox S., Guo G.L., Apte U., Taylor K.M., Sheline C.T., Wang L., Andrews G.K. (2010). Zip4 (Slc39a4) expression is activated in hepatocellular carcinomas and functions to repress apoptosis, enhance cell cycle and increase migration. PLoS ONE.

[57] Gartmann L., Wex T., Grüngreiff K., Reinhold D., Kakinski T., Malfertheiner P., Schütte K. (2018). Expression of zinc transporter ZIP4, ZIP14 and ZnT9 in hepatic carcinogenesis-An immunohistochemical study. J. Trace Elem. Med. Biol..

[58] Li Y., Chen R., Yang J., Mo S., Quek K., Kok C.H., Cheng X.D., Tian S., Zhang W., Qin J.J. (2020). Integrated bioinformatics analysis reveals key candidate genes and pathways associated with clinical outcomes in hepatocellular carcinoma. Front. Genet..

[59] Wang Y.H., Li K.J., Mao L., Hu X., Zhao W.J., Hu A., Lian H.Z., Zheng W.J. (2013). Effects of exogeneous zinc on cell cycle, apoptosis and viability of MDAMB231, HepG2 and 293 T cells. Biol. Trace Elem. Res..

[60] Ye J., Zhang Z., Zhu L., Lu M., Li Y., Zhou J., Lu X., Du Q. (2017). Polaprezinc inhibits liver fibrosis and proliferation in hepatocellular carcinoma. Mol. Med. Rep..

[61] Hashimoto R., Himoto T., Yamada M., Mimura S., Fujita K., Tani J., Morishita A., Masaki T. (2022). Antitumor effect of zinc acetate in hepatocellular carcinoma cell lines via the induction of apoptosis. J. Nutr. Sci. Vitaminol..

[62] El-Desouky M.A., Fahmi A.A., Abdelkader I.Y., Nasraldin K.M. (2020). Anticancer effect of amygdalin (vitamin B17) on hepatocellular carcinoma cell line (HepG2) in the presence and absence of zinc. Anticancer Agents Med. Chem..

[63] Wu R., Mei X., Ye Y., Xue T., Wang J., Sun W., Lin C., Xue R., Zhang J., Xu D. (2019). Zn(II)-curcumin solid dispersion impairs hepatocellular carcinoma growth and enhances chemotherapy by modulating gut microbiota-mediated zinc homeostasis. Pharmacol. Res..

[64] Prasad S., Lall R. (2022). Zinc-curcumin based complexes in health and diseases: An approach in chemopreventive and therapeutic improvement. J. Trace Elem. Med. Biol..

[65] Hassan H.F.H., Mansour A.M., Abo-Youssef A.M.H., Elsadek B.E.M., Messiha B.A.S. (2017). Zinc oxide nanoparticles as a novel anticancer approach; in vitro and in vivo evidence. Clin. Exp. Pharmacol. Physiol..

[66] Matsuoka S., Matsumura H., Nakamura H., Oshiro S., Arakawa Y., Hayashi J., Sekine N., Nirei K., Yamagami H., Ogawa M. (2009). Zinc supplementation improves the outcome of chronic hepatitis C and liver cirrhosis. J. Clin. Biochem. Nutr..

[67] Matsumura H., Nirei K., Nakamura H., Arakawa Y., Higuchi T., Hayashi J., Yamagami H., Matsuoka S., Ogawa M., Nakajima N. (2012). Zinc supplementation therapy improves the outcome of patients with chronic hepatitis, C.. J. Clin. Biochem. Nutr..

[68] Hosui A., Tanimoto T., Okahara T., Ashida M., Ohnishi K., Wakahara Y., Kusumoto Y., Yamaguchi T., Sueyoshi Y., Hirao M. (2021). Oral zinc supplementation decreases the risk of HCC development in patients with HCV eradication by, D.A.A. Hepatol. Commun..

[69] Maret W., Sandstead H.H. (2006). Zinc requirements and the risks and benefits of zinc supplementation. J. Trace Elem. Med. Biol..

[70] Tsang T., Davis C.I., Brady D.C. (2021). Copper biology. Curr. Biol..

[71] Lalioti V., Muruais G., Tsuchiya Y., Pulido D., Sandoval I.V. (2009). Molecular mechanisms of copper homeostasis. Front. Biosci..

[72] Wang Z., Jin D. (2023). Regulatory roles of copper metabolism and cuproptosis in human cancers. Front. Oncol..

[73] Chen L., Min J., Wang F. (2022). Copper homeostasis and cuproptosis in human health and disease. Signal Transduct. Target. Ther..

[74] Tang X., Yan Z. (2023). Copper in cancer: From limiting nutrient to therapeutic target. Front. Oncol..

[75] Hellman N.E., Gitlin J.D. (2002). Ceruloplasmin metabolism and function. Ann. Rev. Nutr..

[76] Arredondo M., Gonzalez M., Olivares M., Pizarro F., Araya M. (2008). Ceruloplasmin, an indicator of copper status. Biol. Trace Elem. Res..

[77] Ward D.M., Kaplan J. (2012). Ferroportin-mediated iron transport: Expression and regulation. Biochem. Biophys. Acta.

[78] Gulec S., Collins J.F. (2014). Molecular mediators govering iron-copper interactions. Ann. Rev. Nutr..

[79] Michalczyk K., Cymbaluk-Ploska A. (2020). The role of zinc and copper in gynecological malignancies. Nutrients.

[80] Gupte A., Mumper R.J. (2009). Elevated copper and oxidative stress in cancer cells as a target for cancer treatment. Cancer Treat. Rev..

[81] Denoyer D., Masaldan S. (2015). Targeting copper in cancer therapy: Copper that cancer. Metallomics.

[82] De Luca A., Barile A., Arciello M., Rossi L. (2019). Copper homeostasis as target of both consolidated and innovative strategies of anti-tumor therapy. J. Trace Elem. Med. Biol..

[83] Han I.W., Jang J.-Y., Kwon W., Park T., Kim Y., Lee K.B., Kim S.-W. (2017). Ceruloplasmin as a prognostic marker in patients with bile duct cancer. Oncotarget.

[84] Zhang Y., Chen Z., Chen J.G., Chen X.F., Gu D.H., Liu Z.M., Gao Y.D., Zheng B. (2021). Ceruloplasmin overexpression is associated with oncogenic pathways and poorer survival rates in clear-cell renal cell carcinoma. FEBS Open Bio.

[85] Voli F., Valli E., Lerra L., Kimpton K., Saletta F., Giorgi F.M., Mercatelli D., Rouaen J.R., Shen S., Murray J.E. (2020). Intratumoral copper modulates PD-L1 expression and influences tumor immune evasion. Cancer Res..

[86] Tsvetkov P., Coy S., Petrova B., Dreishpoon M., Verma A., Abdusamad M., Rossen J., Joesch-Cohen L., Humeidi R., Spangler R.D. (2022). Copper indices cell death by targeting lipoylated TCA cycle proteins. Science.

[87] Yang W., Wang Y., Huang Y., Yu J., Wang T., Li C., Yang L., Zhang P., Shi L., Yin Y. (2023). 4-octyl itaconate inhibits aerobic glycolysis by targeting GAPDH to promote cuproputosis in colorectal cancer. Bio Med. Pharmacother..

[88] Ishida S., McCormick F., Smith-McCune K., Schlatter E. (2010). Enhancing tumor-specific uptake of anticancer drug cisplatin with a copper chelator. Cancer Cell.

[89] Lee Y.-Y., Choi C.H., Do I.-G., Song S.Y., Lee W., Park H.S., Song T.J., Kim M.K., Kim T.-J., Lee J.-W. (2011). Prognostic value of the copper transporters, CTR1 and CTR2, in patients with ovarian carcinoma receiving platinum-based chemotherapy. Gynecol. Oncol..

[90] Shanbhag V.C., Gudekar N., Jasmer K., Papageorgiou C., Singh K., Petris M.J. (2021). Copper metabolism as a unique vulnerability in cancer. Biochim. Biophys. Acta Mol. Cell Res..

[91] Komatsu M., Sumizawa T., Mutoh M., Chen Z.S., Terada K., Furukawa T., Yang X.L., Gao H., Miura N., Sugiyama T. (2000). Copper-transporting P-type adenosine triphosphatase (ATP7B) is associated with cisplatin resistance. Cancer Res..

[92] Sugeno H., Takebayashi Y., Higashimoto M., Ogura Y., Shibukawa G., Kanzaki A., Terada K., Sugiyama T., Watanabe K., Katoh R. (2004). Expression of copper-transporting P-type adenosine triphosphatase (ATP7B) in human hepatocellular carcinoma. Anticancer Res..

[93] Porcu C., Antonucci L., Barbaro B., Illi B., Nasi S., Martini M., Licata A., Miele L., Grieco A., Balsano C. (2018). Copper/MYC/CTR1 interplay: A dangerous relationship in hepatocellular carcinoma. Oncotarget.

[94] Iwadate H., Ohira H., Suzuki T., Abe K., Yokokawa J., Takiguchi J., Rai T., Orikasa H., Irisawa A., Obara K. (2004). Hepatocellular carcinoma associated with Wilson’s disease. Intern. Med..

[95] Pfeiffenberger J., Mogler C., Gotthardt D.N., Schulze-Bergkamen H., Litwin T., Reuner U., Hefter H., Huster D., Schemmer P., Członkowska A. (2015). Hepatobiliary malignancies in Wilson disease. Liver Int..

[96] Poo J.L., Rosas-Romero R., Montemayor A.C., Isoard F., Uribe M. (2003). Diagnostic value of copper/zinc ratio in hepatocellular carcinoma: A case control study. J. Gastroenterol..

[97] Feng Y., Zeng J.W., Ma Q., Zhang S., Tang J., Feng J.F. (2020). Serum copper and zinc levels in breast cancer: A meta-analysis. J. Trace Elem. Med. Biol..

[98] Zhang L., Shao J., Tan S.W., Ye H.P., Shan X.Y. (2022). Association between serum copper/zinc ratio and lung cancer: A systematic review with meta-analysis. J. Trace Elem. Med. Biol..

[99] Tamai Y., Iwasa M., Eguchi A., Shigefuku R., Sugimoto K., Hasegawa H., Takei Y. (2020). Serum copper, zinc metallothionein serve as potential biomarker for hepatocellular carcinoma. PLoS ONE.

[100] Ebara M., Fukuda H., Hatano R., Saisho H., Nagato Y., Suzuki K., Nakajima K., Yukawa M., Kondo F., Nakayama A. (2000). Relationship between copper, zinc and metallothionein in hepatocellular carcinoma and its surrounding liver parenchyma. J. Hepatol..

[101] Casaril M., Capra F., Marchiori L., Gabrielli G.B., Nicoli N., Corso F., Baracchino F., Corrocher R. (1989). Serum copper and ceruloplasmin in early and in advanced hepatocellular carcinoma: Diagnostic and prognostic relevance. Tumori.

[102] Ferrin G., Rodriguez-Perálvarez M., Aguilar-Melero P., Ranchal I., Liamoza C., Linares C. (2015). Plasma protein biomarkers of hepatocellular carcinoma in HCV-infected alcoholic patients with cirrhosis. PLoS ONE.

[103] Yin H., Lin Z., Nie S., Wu J., Tan Z., Zhu J., Dai J., Feng Z., Marrero J., Lubman D.M. (2014). Mass-selected site-specific core-fucosylation of ceruloplasmin in alcohol-related hepatocellular carcinoma. J. Proteome Res..

[104] Gan B. (2021). Mitochondrial regulation of ferroptosis. J. Cell Biol..

[105] Shang Y., Luo M., Yao F., Wang S., Yuan Z., Yang Y. (2020). Ceruloplasmin suppresses ferroptosis by regulating iron homeostasis in hepatocellular carcinoma cells. Cell Signal.

[106] Wang X., Chen D., Shi Y., Luo J., Zhang Y., Yuan X., Zhang C., Shu H., Yu W., Tian J. (2023). Copper and cuproptosis-related genes in hepatocellular carcinoma: Therapeutic biomarkers targeting tumor immune microenvironment and immune checkpoints. Front. Immunol..

[107] Lin D., Wu J. (2015). Hypoxia inducible factor in hepatocellular carcinoma: A therapeutic target. World J. Gastroenterol..

[108] Himoto T., Fujita K., Nomura T., Tani J., Miyoshi H., Morishita A., Yoneyama H., Kubota S., Haba R., Suzuki Y. (2016). Roles of copoper in hepatocarcinogenesis via the activation of hypoxia-inducible factor-1a. Biol. Trace Elem. Res..

[109] Sone K., Maeda M., Wakabayashi K., Takeichi N., Mori M., Sugimura T., Nagao M. (1996). Inhibition of hereditary hepatitis and liver tumor development in Long-Evans cinnamon rats by the copper-chelating agent trientine dihydrochloride. Hepatology.

[110] Yoshii J., Yoshiji H., Kuriyama S., Ikenaka Y., Noguchi R., Okuda H., Tsujinoue H., Nakatani T., Kishida H., Nakae D. (2000). The copper-chelating agent, trientine, suppresses tumor development and angiogenesis in the murine hepatocellular carcinoma cells. Int. J. Cancer.

[111] Moriguchi M., Nakajima T., Kimura H., Watanabe T., Takashima H., Mitsumoto Y., Katagishi T., Okanoue T., Kagawa K. (2002). The copper chelator trientine has an antiangiogenic effect against hepatocellular carcinoma, possibly through inhibition of interleukin-8 production. Int. J. Cancer.

[112] Davis C.I., Gu X., Kiefer R.M., Ralle M., Gade T.P., Brady D.C. (2020). Altered copper homeostasis underlies sensitivity of hepatocellular carcinoma to copper chelation. Metallomics.

[113] Li Y., Fu S.Y., Wang L.H., Wang F.Y., Wang N.N., Cao Q., Wang Y.T., Yang J.Y., Wu C.F. (2015). Copper improves the anti-angiogenic activity of dusulfiram through the EGFR/Src/VEGF pathway in glioma. Cancer Lett..

[114] Li Y., Wang L.H., Zhang H.T., Wang Y.T., Liu S., Zhou W.L., Yuan X.Z., Li T.Y., Wu C.F., Yang J.Y. (2018). Disulfiram combined with copper inhibits metastasis and epithelial-mesenchymal transition in hepatocellular carcinoma through the NF-kB and TGF-b pathways. J. Cell Mol. Med..

[115] Llovet J.M., Ricci S., Mazzaferro V., Hilgard P., Gane E., Blanc J.F., De Oliveira A.C., Santoro A., Raoul J.L., Forner A. (2008). Sorafenib in advanced hepatocellular carcinoma. N. Engl. J. Med..

[116] Ren X., Li Y., Zhou Y., Hu W., Yang C., Jing Q., Zhou C., Wang X., Hu J., Wang L. (2021). Overcoming the compensatory elevation of NRF2 renders hepatocellular carcinoma cells more vulnerable to disulfiram/copper-induced ferroptosis. Redox Biol..

[117] Zhou B., Guo L., Zhang B., Liu S., Zhang K., Yan J., Zhang W., Yu M., Chen Z., Xu Y. (2019). Disulfiram combined with copper induces immunosuppression via PD-L1 stabilization in hepatocellular carcinoma. Am. J. Cancer Res..

[118] Gao F., Yuan Y., Ding Y., Li P.Y., Chang Y., He X.X. (2023). DLAT as a cuproptosis promotor and molecular target of *elesclomol* in hepatocellular carcinoma. Curr. Med. Sci..

[119] Li D., Shi Z., Liu X., Jin S., Chen P., Zhang Y., Chen G., Fan X., Yang J., Lin H. (2023). Identification and development of a novel risk model based on cuproptosis-associated RNA methylation regulators for predicting prognosis and characterizing immune status in hepatocellular carcinoma. Hepatol. Int..

[120] Fu Z.G., Wang L., Cui H.Y., Peng J.L., Wang S.J., Geng J.J., Feng F., Song F., Li L., Zhu P. (2016). A novel small-molecule compound targeting CD147 inhibits the motility and invasion of hepatocellular carcinoma cells. Oncotarget.

[121] Yan L., Zucker S., Toole B.P. (2005). Roles of the multifunctional glycoprotein, emmprin (basigin; CD147), in tumour progression. Tromb. Haemost..

[122] Qin C., Liu H., Chen K., Hu X., Ma X., Lan X., Zhang Y., Cheng Z. (2014). Theranostics of malignant melanoma with ^64^CuCl_2_. J. Nucl. Med..

[123] Wachsmann J., Peng F. (2016). Molecular imaging and therapy targeting copper metabolism in hepatocellular carcinoma. World J. Gastroenterol..

[124] Siddiqui M.A., Alhadlaq H.A., Ahmad J., Al-Khedhairy A.A., Musarrat J., Ahamed M. (2013). Copper oxide nanoparticles induced mitochondria mediated apoptosis in human hepatocarcinoma cells. PLoS ONE.

[125] Brewer G.J., Dick R.D., Grover D.K., LeClaire V., Tseng M., Wicha M., Pienta K., Redman B.G., Jahan T., Sondak V.K. (2000). Treatmentof metastatic cancer with tetrathiomolybdate, an anticopper, antiangiogenetic agent: Phase I study. Clin. Cancer Res..

[126] Redman B.G., Esper P., Pan Q., Dunn R.L., Hussain H.K., Chenevert T., Brewer G.J., Merajver S.D. (2003). Phase II trial of trtrathiomolybdate in patients with advanced kidney cancer. Clin. Cancer Res..

[127] Ohkoshi-Yamada M., Kamimura K., Kamimura H., Terai S. (2021). Rare complication of hepatocellular carcinoma in Wilson’s disease. JGH Open.

[128] Rayman M.P. (2000). The importance of selenium to human health. Lancet.

[129] Roman M., Jitaru P., Barbante C. (2014). Selenium biochemistry and its role for human health. Metallomics.

[130] Razaghi A., Poorebrahim M., Sarhan D., Björnstedt M. (2021). Selenium stimulates the antitumor immunity: Insights to future research. Eur. J. Cancer.

[131] Zeng H., Combs G.F. (2008). Selenium as an antitumor nutrient: Roles in cell proliferation and tumor invasion. J. Nutr. Biochem..

[132] Yildiz A., Kaya Y., Tanriverdi D. (2019). Effect of interaction between selenium and zinc on DNA repair in association with cancer prevention. J. Cancer Prev..

[133] Lee K.H., Jeong D. (2012). Bimodal actions of selenium essential for antioxidant and toxic pro-oxidant activities: The selenium paradox(review). Mol. Med. Rep..

[134] Zeng H. (2002). Selenite and selenomethionine promote HL-60 cell cycle progression. J. Nutr..

[135] Brozmanová J., Mániková D., Vlčková V., Chovanec M. (2010). Selenium: A double-edged sword for defense and offence in cancer. Arch. Toxicol..

[136] Wallenberg M., Misra S., Björnstedt M. (2014). Selenium cytotoxicity in cancer. Basic. Clin. Pharmacol. Toxicol..

[137] Fernandes A., Gandin V. (2015). Selenium compounds as therapeutic agents in cancer. Biochim. Biophys. Acta.

[138] Thirunavukkarasu C., Premkumar K., Sheriff A.K., Sakthisekaran D. (2008). Sodium selenite enhances glutathione peroxidase activity and DNA single strand breaks in hepatoma induced by N-nitrosodiethylamine and promoted by Phenobarbital. Mol. Cell Biochem..

[139] Rudolf E., Rudolf K., Červinka M. (2008). Selenium activates p53 and p38 pathways and induces caspase-independent cell death in cervical cancer cells. Cell Biol. Toxicol..

[140] Li Z., Meng J., Xu T.J., Qin X.Y., Zhou X.D. (2013). Sodium selenite induces apoptosis in colon cancer cells via Bax-dependent mitochondrial pathway. Eur. Rev. Med. Pharmacol. Sci..

[141] Corcoran N.M., Najdovska M., Costello A.J. (2004). Inogranic selenium retards progression of experimental hormone refractory prostate cancer. J. Urol..

[142] Kiremidjian-Schumacher L., Roy M., Glickman R., Schneider K., Rothstein S., Cooper J., Hochster H., Kim M., Newman R. (2000). Selenium and immunocompetence in patients with head and neck cancer. Biol. Trace Elem. Res..

[143] Avery J.C., Hoffmann P.R. (2018). Selenium, selenoprotein and immunity. Nutrients.

[144] Lai H., Zeng D., Liu C., Zhang Q., Wang X., Chen T. (2019). Selenium containing ruthenium complex synergizes with natural killer cells to enhance immunotherapy against prostate cancer via activating TRAIL/FasL signaling. Biomaterials.

[145] Wang J., Chang Y., Luo H., Jiang W., Xu L., Chen T., Zhu X. (2020). Designing immunogenic nanotherapeutic for photothermal-triggered immunotherapy involving reprogramming immunosuppression and activating systemic antitumor responses. Biomaterials.

[146] Meyer F., Galan P., Douville P., Bairati I., Kegle P., Bertrais S., Estaquio C., Hercberg S. (2005). Antioxidant vitamin and mineral supplementation and prostate cancer prevention in the SU.VI.MAX trial. Int. J. Cancer.

[147] Yang W.S., SriRamaratnam R., Welsch M.E., Shimada K., Skouta R., Viswanathan V.S., Cheah J.H., Clemons P.A., Shamji A.F., Clish C.B. (2014). Regulation of ferroptotic cancer cell death by GPX4. Cell.

[148] Choi J.A., Lee E.H., Cho H., Kim J.H. (2023). High dose of selenium induces ferroptotic cell death in ovarian cancer. Int. J. Mol. Sci..

[149] Kalimuthu K., Keerthana C.K., Mohan M., Arivalagan J., Christyraj J.R.S.S., Firer M.A., Choudry M.H.A., Anto R.J., Lee Y.J. (2022). Emerging role of selenium metabolic pathways in cancer: New therapeutic target for cancer. J. Cell Biochem..

[150] Zhang X.-D., Liu Z.-Y., Wang M.-S., Guo Y.-X., Wang X.-K., Luo K., Huang S., Li R.-F. (2023). Mechanisms and regulations of ferroptosis. Front. Immunol..

[151] Liu Z., Yang W., Long G., Wei C. (2016). Trace elements and chemotherapy sensitivity. Biol. Trace Elem. Res..

[152] Abd-Rabou A.A., Shalby A.B., Ahmed H.H. (2019). Selenium nanoparticle induce the chemosensitivity of fluorouracil nanoparticles in breast and colon cancer cells. Biol. Trace Elem. Res..

[153] Varlamova E.G., Khabatova V.V., Gudkov S.V., Turovsky E.A. (2023). Ca^2+^-dependent effects of selenium-sorafenib nanocomplexs on glioblastoma cells and astrocytes of the cerebral cortex: Anticancer agent and cytoprotector. Int. J. Mol. Sci..

[154] Himoto T., Yoneyama H., Kurokohchi K., Inukai M., Masugata H., Goda F., Haba R., Watababe S., Kubota S., Senda S. (2011). Selenium deficiency is associated with insulin resistance in patients with hepatitis C virus-related chronic liver disease. Nutr. Res..

[155] Bettinger D., Schultheiss M., Hennecke N., Panther E., Knüppel E., Blum H.E., Thimme R., Spangenberg H.C. (2013). Selenium levels in patients with hepatitis C virus-related chronic hepatitis, liver cirrhosis, and hepatocellular carcinoma: A pilot study. Hepatology.

[156] Kim I.W., Bae S.M., Kim Y.W., Liu H.B., Bae S.H., Choi J.Y., Yoon S.K., Chaturvedi P.K., Battogtokh G., Ahn W.S. (2012). Serum selenium levels in Krean hepatoma patients. Biol. Trace Elem..

[157] Hughes D.J., Duarte-Salles T., Hybsier S., Trichopoulou A., Stepien M., Aleksandrova K., Overvad K., Tjønneland A., Olsen A., Affret A. (2016). Prediagnostic selenium status and hepatobiliary cancer risk in the European prospective investigation into cancer and nutrition cohort. Am. J. Clin. Nutr..

[158] Zhang Z., Bi M., Liu Q., Yang J., Xu S. (2016). Meta-analysis of the correlation between selenium and incidence of hepatocellular carcinoma. Oncotarget.

[159] Gong Y., Dong F., Geng Y., Zhuang H., Ma Z., Zhou Z., Huang B., Sun Z., Hou B. (2019). Selenium concentration, dietary intake and risk of hepatocellular carcinoma-A systemic review with meta-analysis. Nutr. Hosp..

[160] Rohr-Udilova N., Sieghart W., Eferl R., Stoiber D., Björkhem-Bergman L., Eriksson L.C., Stolze K., Hayden H., Keppler B., Sagmeister S. (2012). Antagonistic effects of selenium and lipid peroxides on growth control in early hepatocellular carcinoma. Hepatology.

[161] Stasio M.D., Volpe M.G., Colonna G., Nazzaro M., Polimeno M., Scala S., Castello G., Costantini S. (2011). A possible predictive marker of progression for hepatocellular carcinoma. Oncol. Lett..

[162] Rohr-Udilova N., Bauer E., Timelthaler G., Eferl R., Stolze K., Pinter M., Seif M., Hayden H., Reiberger T., Schulte-Hermann R. (2018). Impact of glutathione peroxidase 4 on cell proliferation, angiogenesis and cytokine production in hepatocellular carcinoma. Oncotarget.

[163] Alves A.D., Moura A.C., Andreolla H.F., Veiga A.B., Fiegenbaum M., Giovenardi M., Almeida S. (2021). Gene expression evaluation of antioxidant enzymes in patients with hepatocellular carcinoma: RT-qPCR and bioinformatic analyses. Genet. Mol. Biol..

[164] Guerriero E., Capone F., Accardo M., Sorice A., Costantini M., Colonna G., Castello G., Costantini S. (2015). GPX4 and GPX7 over-expression in human hepatocellular carcinoma tissues. Eur. J. Histochem..

[165] Radomska D., Czarnomysy R., Radomski D., Bielawska A., Bielawski K. (2021). Selenium as a bioactive micronutrient in the human diet and its cancer chemopreventive activity. Nutrients.

[166] Elhodaky M., Diamond A.M. (2018). Selenium-binding protein 1 in human health and disease. Int. J. Mol. Sci..

[167] Corona G., De Lorenzo E., Elia C., Simula M.P., Avellini C., Baccarani U., Lupo F., Tiribelli C., Colombatti A., Toffoli G. (2010). Differential proteomic analysis of hepatocellular carcinoma. Int. J. Oncol..

[168] Huang C., Ding G., Gu C., Zhou J., Kuang M., Ji Y., He Y., Kondo T., Fan J. (2012). Decreased selenium-binding protein 1 enhances glutathione peroxidase 1 activity and downregulates HIF-1a to promote hepatocellular carcinoma invasiveness. Clin. Cancer Res..

[169] Gao P.T., Ding G.Y., Yang X., Dong R.Z., Hu B., Zhu X.D., Cai J.B., Ji Y., Shi G.M., Shen Y.H. (2018). Invasive potential of hepatocellular carcinoma is enhanced by loss of selenium-binding protein 1 and subsequent upregulation of CXCR4. Am. J. Cancer Res..

[170] Sutton A., Nahon P., Pessayre D., Rufat P., Poiré A., Ziol M., Vidaud D., Barget N., Ganne-Carrié N., Charnaux N. (2006). Genetic polymorphisms in antioxidant enzymes modulated hepatic iron accumulation and hepatocellular carcinoma development in patients with alcohol-induced cirrhosis. Cancer Res..

[171] Lincoln D.T., Ali Emadi E.M., Torissen K.F., Clarke F.M. (2003). The thioredoxin-thioredoxin reductase system: Over-expression in human cancer. Anticancer Res..

[172] Fu B., Meng W., Zeng X., Zhao H., Liu W., Zhang T. (2017). TXNR1 is an unfavorable prognostic factor for patients with hepatocellular carcinoma. BioMed Res. Int..

[173] Lee D., Xu I.M., Chiu D.K., Leibold J., Tse A.P., Bao M.H., Yuen V.W., Chan C.Y., Lai R.K., Chin D.W. (2019). Induction of oxidative stress via inhibition of thioredoxin reductase 1 is an effective therapeutic approach for hepatocellular carcinoma. Hepatology.

[174] Li C., Peng Y., Mao B., Qian K. (2015). Thioredoxin reductase: A novel, independent prognostic marker in patients with hepatocellular carcinoma. Oncotarget.

[175] Hua S., Quan Y., Zhan M., Liao H., Li Y., Lu L. (2019). miR-125-5p inhibits cell proliferation, migration, and invasion in hepatocellular carcinoma via targeting TXNRD1. Cancer Cell Int..

[176] Celik H.A., Aydin H.H., Deveci R., Terzioglu E., Karacali S., Saydam G., Akarca U., Batur Y. (2004). Biochemical and morphological characteristics of selenite-induced apoptosis in human hepatoma HepG2 cells. Biol. Trace Elem. Res..

[177] Borda L.J., Perper M., Keri J.E. (2019). Treatment of seborrheic dermatitis: A comprehensive review. J. Dermatol. Treat..

[178] Yang T., Huo J., Xu R., Su Q., Tang W., Zhang D., Zhu M., Zhan Y., Dai B., Zhang Y. (2021). Selenium sulfide disrupts the PLAGL2/C-MET/STAT3-induced resistance against mitochondrial apoptosis in hepatocellular carcinoma. Clin. Transl. Med..

[179] Cuello S., Ramos S., Mateos R., Martín M.A., Madrid Y., Cámara C., Bravo L., Goya L. (2007). Selenium methylselenocysteine protects human hepatoma HepG2 cells against oxidative stress induced by tert-butyl hydroperoxide. Anal. Bilanal. Chem..

[180] Liu J.G., Zhao H.J., Liu Y.J., Liu Y.W., Wang X.L. (2012). Effect of two selenium sources on hepatocarcinogenesis and several angiogenic cytokines in diethylnitrosamine-induced hepatocarcinoma rats. J. Trace Elem. Med. Biol..

[181] Mao G., Li Q., Deng C., Wang Y., Ding Y., Zhang W., Chen Y., Zhao T., Wei F., Yang L. (2018). The synergism and attenuation effect of selenium (Se)-enriched Grifola frondose-polysaccharide on 5-fluorouracil (5-Fu) in Heps-bearing mice. Int. J. Biol. Macromol..

[182] Khurana A., Tekula S., Saifi M.A., Venkatesh P., Godugu C. (2019). Therapeutic application of selenium nanoparticles. Biomed. Pharmacother..

[183] Singh D., Singh M. (2021). Hepatocellular-targeted mRNA delivery using functionalized selenium nanoparticles in vitro. Pharmaceutics.

[184] Xia Y., Zhong J., Zhao M., Tang Y., Han N., Hua L., Xu T., Wang C., Zhu B. (2019). Galactose-modified selenium nanoparticles for targeted delivery of doxorubicin to hepatocellular carcinoma. Drug Deliv..

[185] Al-Noshokaty T.M., Mesbah N.M., Abo-Elmatty D.M., Abulsoud A.I., Abdel-Hamed A.R. (2022). Selenium nanoparticles overcome sorafenib resistance in thioacetamide induced hepatocellular carcinoma in rats by modulation of mTOR, NF-kB pathways and LncRNA-AF085935/GPC3 axis. Life Sci..

[186] Zheng X., Ma W., Sun R., Yin H., Lin F., Liu Y., Xu W., Zeng H. (2018). Butaselen prevents hepatocarcinogenesis and progression through inhibiting thioredoxin reductase activity. Redox Biol..

[187] Zhang Q., Chen W., Lv X., Weng Q., Chen M., Cui R., Liang G., Ji J. (2019). Piperlongumine, a novel TrxR1 inhibitor, induces apoptosis in hepatocellular carcinoma cells by ROS-mediated ER stress. Front. Pharmacol..

[188] Su X., Yin H., Bai M., Liu J., Liu R., Zeng H., Wen J. (2023). A novel TrxR1 inhibitor regulates NK and CD8+ T cell infiltration and cytotoxicity, enhancing the efficacy of anti-PD-1 immunotherapy against hepatocellular carcinoma. J. Immunol..

